# Death to Small Publishers

**Published:** 1896-04

**Authors:** 


**Death to Small Publishers.**—A Mr. Loud, of California, has introduced a bill into congress, which, if it should become a law, will simply amount to prohibition, as far as small publications are concerned. It is a most iniquitous scheme, and every man interested in the publication business should co-operate in securing its defeat. The following circular, which has been sent to all periodicals, will explain the case fully, and is reproduced here as an item of news of interest, not only to publishers, but to readers. Let every publisher write to his immediate representatives in congress a strong protest, and ask their influence to defeat the scheme:

NEW YORK, March 7th, 1896.

*To the Publisher Texas Medical Journal:*

DEAR SIR:—We beg to call your attention to the enclosed copy of a bill, known as H. R. 4566, relating to second-class matter, introduced by Mr. Loud, of California, and now upon the calendar of the House of Representatives.

The bill is most sweeping in its provisions, and calculated, should it become a law, to work great hardship and dire disaster to hundreds of thousands of persons engaged in the publishing, printing, paper-making and allied trades, as well as to the public at large.

The following are some of its objectionable features:

1. It excludes from the mails as second-class matter all "books or reprints of books," by which is meant all paper-covered books issued periodically, which have done so much to popularize cheap and good literature among the masses of the people, placing the choicest works of literature, and of science, within the reach of the humblest schoolboy, and according to our people, an intellectual privilege, such as was never enjoyed by the citizens of any other country. The bill proposes to crush this class of literature most completely and effectually.

2. It excludes from the mails as second-class matter all "sample copies" of newspapers and periodicals, thus depriving all publications of one of the most valuable methods, not only of extending their circulations, but of securing new subscribers to take the places of those who die or drop out for various causes. It, therefore, means the general depletion of newspaper circulations.

3. It increases the rate of postage upon "returns" to news agents from one cent to four cents per pound, thus seriously crippling the circulation of that large class of periodicals that are sent out "on sale."

4. It permits the mailing of periodicals at the pound rate to subscribers only, and defines a subscriber as one who "voluntarily orders and pays for the same." Under this definition a person whose subscription has lapsed, and has not been renewed, is not a subscriber, and copies of a periodical sent to other than advance-paying subscribers could be excluded. This is a direct blow at the local country newspaper.

5. It requires publishers who are permitted to mail matter of the second-class to separate the same, before mailing, into United States mail sacks, or bundles, by States, cities, towns and counties, as the postmaster-general may direct, thus forcing every publisher to establish in his office a miniature postoffice, and entailing great extra expense.

6. It is estimated that the enactment of this measure will reduce the consumption of white paper to the extent of 100,000 tons annually. That means 100,000 tons less of paper to be made, so much less printing, type-setting, electrotyping and binding. It means that hundreds of thousands of working men and women will be thrown out of employment, entailing distress and calamity, the extent of which can hardly be estimated.

7. The advocates of the bill maintain that its purpose is to reduce the deficiency in tne postal revenues. They claim that there is a heavy loss to the government in the carriage of second-class matter. This claim is untenable, because whatever loss there may be upon the carriage of second-class matter is fully offset by the gain upon the carriage of two profitable classes, the first and fourth, which are a direct outgrowth of the mailing of second-class matter. Every publisher receives large numbers of letters, and mails large quantities of "premiums" (fourth-class matter) to his subscribers and club-raisers, and almost every advertisement is a bid for correspondence by mail, and the consequent mailing of merchandise in filling orders. There are other causes than second-class matter to account for the annual deficiency in the postal revenues, which would still be operative should the bill become a law. How, then, could the enactment of this measure reduce the deficiency?

8. The real beneficiaries of the measure are the express companies. By cutting off competition with the postal service, they would be enabled to increase their rates, and thus reap a rich harvest.

We need your help to defeat this obnoxious measure. If you will sign the enclosed protest and send it to the member from your district, or, better still, if you will write a letter of similar tenor and send it to each member of the House from your section, and to the two senators from your state, and if you will publish an editorial opposing the bill, and send a marked copy of your paper containing it to each senator and representative from your section, great good will undoubtedly result. Thanking you in advance for whatsoever favors you may render to us in this connection, we remain, Very truly yours,

John Elderkin,
O. J. Victor,
T. A. Vernon,
Committee of New York Publishers.

